# Peroxidasin Inhibition by Phloroglucinol and Other Peroxidase Inhibitors

**DOI:** 10.3390/antiox13010023

**Published:** 2023-12-21

**Authors:** Martina Paumann-Page, Christian Obinger, Christine C. Winterbourn, Paul G. Furtmüller

**Affiliations:** 1 Mātai Hāora Centre for Redox Biology and Medicine, University of Otago Christchurch, Ōtautahi Christchurch 8011, New Zealand; christine.winterbourn@otago.ac.nz; 2 Institute of Biochemistry, Department of Chemistry, University of Natural Resources and Life Sciences, Vienna, Muthgasse 18, 1190 Vienna, Austria; christian.obinger@boku.ac.at

**Keywords:** peroxidasin, inhibition of catalytic activity, phloroglucinol, peroxidase inhibitors, hypobromous acid, oxidative stress, myeloperoxidase, eosinophil peroxidase, lactoperoxidase, thyroid peroxidase

## Abstract

Human peroxidasin (PXDN) is a ubiquitous peroxidase enzyme expressed in most tissues in the body. PXDN represents an interesting therapeutic target for inhibition, as it plays a role in numerous pathologies, including cardiovascular disease, cancer and fibrosis. Like other peroxidases, PXDN generates hypohalous acids and free radical species, thereby facilitating oxidative modifications of numerous biomolecules. We have studied the inhibition of PXDN halogenation and peroxidase activity by phloroglucinol and 14 other peroxidase inhibitors. Although a number of compounds on their own potently inhibited PXDN halogenation activity, only five were effective in the presence of a peroxidase substrate with IC_50_ values in the low μM range. Using sequential stopped-flow spectrophotometry, we examined the mechanisms of inhibition for several compounds. Phloroglucinol was the most potent inhibitor with a nanomolar IC_50_ for purified PXDN and IC_50_ values of 0.95 μM and 1.6 μM for the inhibition of hypobromous acid (HOBr)-mediated collagen IV cross-linking in a decellularized extracellular matrix and a cell culture model. Other compounds were less effective in these models. Most interestingly, phloroglucinol was identified to irreversibly inhibit PXDN, either by mechanism-based inhibition or tight binding. Our work has highlighted phloroglucinol as a promising lead compound for the design of highly specific PXDN inhibitors and the assays used in this study provide a suitable approach for high-throughput screening of PXDN inhibitors.

## 1. Introduction

Peroxidasin (PXDN) is an evolutionary conserved multidomain peroxidase secreted to the basement membrane (BM) [1,2]. PXDN catalyses the oxidation of bromide to hypobromous acid (HOBr) to generate a covalent cross-link in the non-collagenous domain (NCD) of collagen IV [3,4]. This specific sulfilimine bond contributes to basement membrane stability and its biophysical properties [5,6,7,8]. However, HOBr and secondary oxidation products like bromamines are reactive oxidants, known for their harmful effects on a multitude of biomolecules and their implication in diseases [9,10,11,12,13]. The detection of bromotyrosine, a stable product of the reaction of HOBr with tyrosine, emphasizes that HOBr generated by PXDN leads to the oxidation of extracellular matrix (ECM) proteins and, to a lesser extent, intracellular proteins [12,14,15].

There is increasing evidence that PXDN plays a role in several pathologies. PXDN was shown to play an adverse role in various cardiovascular conditions including hypertension-associated endothelial dysfunction [16,17,18], vascular remodelling and calcification [19], cardiac hypertrophy [18], atherosclerosis [20,21,22], aortic aneurysms [23], ischemic cardiac injury and fibrosis [24], as well as pulmonary arterial hypertension [25]. PXDN is upregulated in tissue fibrosis [2,26] and contributes to the progression of numerous types of cancer [27,28,29,30,31,32]. PXDN activity was shown to promote angiogenesis [33] and PXDN expression is associated with an increased invasive potential of cancer cells [29], possibly due to the remodelling of the tumour ECM to promote a permissive environment for cell invasion and metastasis. 

The detailed underlying mechanisms are not well understood, but dysregulated oxidant generation and subsequent maladaptive cell signalling, that promotes oxidative stress, inflammation, extracellular matrix remodelling and endothelial dysfunction, is a common working hypothesis. For this reason, the pharmacological inhibition of PXDN would be beneficial, to reduce disease progression that is attributed to its catalytic activity [34,35]. 

**Figure 1:**
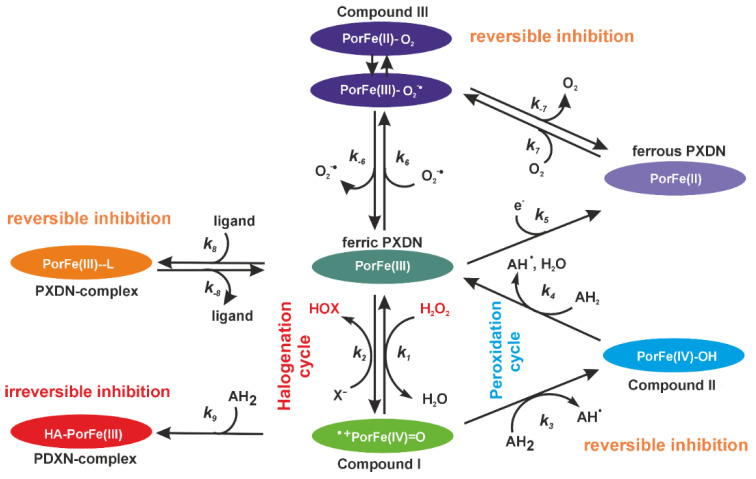
Scheme of halogenation and peroxidation cycle of PXDN. Reaction 1: native ferric PXDN is oxidized by hydrogen peroxide to Compound I (oxoiron (IV) porphyryl radical). In reaction 2, Compound I is directly reduced back to the native state by bromide, iodide or thiocyanate [46], releasing the respective hypohalous acid (HOX). Reactions 1 and 2 constitute the halogenation cycle. In reaction 3, Compound I is reduced to Compound II via a one-electron reduction. In reaction 4, Compound II is reduced to the native form of PXDN by a second one-electron donor (AH_2_, i.e., ascorbate, tyrosine, serotonin, nitrite or urate [14,41]). Reactions 1, 3 and 4 constitute the peroxidase cycle. Compound III forms from ferric or ferrous PXDN with superoxide (reaction 6) or dioxygen (reaction 7), respectively. Reaction 5 entails the reduction of ferric to ferrous PXDN by an electron donor. A good PXDN inhibitor should either: (i) efficiently block the entry to the reactive site (reaction 8, reversible); (ii) promote accumulation of Compound II or Compound III, which are outside of the halogenation cycle (reactions 3, 5 or 6, reversible); lead to (iii), a complex formation with the active site (reaction 8, reversible); or (iv) a covalent irreversible adduct formation (reaction 9, irreversible).

PXDN belongs to family 2 of the peroxidase–cyclooxygenase superfamily [36,37]. As well as the catalytically active peroxidase domain, PXDN also comprises a leucine-rich repeat (LRR) domain, four C-like immunoglobulin (Ig) domains at the N terminus and a C-terminal von Willebrand factor type C module (VWC). Mature PXDN is highly glycosylated and forms homotrimers via intermolecular disulfide bonds [8,38,39]. The peroxidase domain has high sequence homology to the well-characterized chordata peroxidases myeloperoxidase (MPO), lactoperoxidase (LPO), eosinophil peroxidase (EPO) and thyroid peroxidase (TPO). Figure 1 depicts a reaction scheme with PXDN in its resting ferric state in the centre. PXDN catalyses the hydrogen peroxide-mediated oxidation of halides (halogenation cycle) and peroxidase substrates (peroxidation cycle). Compound III is a further redox intermediate that can be formed via ferrous PXDN or when ferric PXDN reacts with superoxide.

HOBr is the most potent oxidant generated by PXDN and there are different strategies to inhibit its formation. Inhibitors which are substrates of the peroxidation cycle are able to modulate PXDN halogenation reactions by promoting the formation of halogenation-inactive Compound II [40,41] or Compound III [42]. However, these types of inhibition are reversible and may be short-lived [43]. Another form of reversible inhibition occurs when an inhibitor binds tightly to ferric PXDN, thereby blocking the active site from reacting with any substrates [44]. Irreversible inhibition occurs when an inhibitor forms a covalent adduct and renders the enzyme inactive [45]. This type of inhibition is referred to as mechanism-based inhibition by a suicide substrate and represents the most effective way of irreversible enzyme inhibition (Figure 1). 

For the rationale design of highly specific inhibitors, in silico docking and modelling can be used to screen large drug libraries, as previously shown for MPO [47]. However, these methods depend on a high-resolution structure, which is not available for PXDN. Nevertheless, a homology model of PXDN, based on the structure of LPO, confirms the high similarities of the PXDN peroxidase domain with LPO and recently resolved small-angle X-ray scattering (SAXS)-X-ray hybrid structures, giving an indication of the overall three-dimensional arrangement of the three monomers in the trimer structure and of the location of the additional domains of PXDN (LRR, Ig and peroxidase domain (POX)) [39]. Inhibitors of other peroxidases can therefore be used as a guide for PXDN inhibitor design.

In particular, MPO, and to a lesser extent LPO and EPO, have been studied extensively and great efforts have been made in the rationale design of specific inhibitors for these peroxidases [48,49]. Based on the high homology of the active site structure that mammalian peroxidases share with each other, it is likely that an inhibitor does not just inhibit one but also other peroxidases. Hence, we have investigated the inhibition of PXDN by potent MPO and EPO inhibitors, as well as phloroglucinol, which has been shown to inhibit PXDN [3,4,14,29,50,51] potently but also TPO and LPO [52]. Table 1 summarizes the compounds used in this study. Phloroglucinol, lomefloxacin, thioridazine, metoclopramide and hydralazine-vanillin are approved drugs and the latter four were used in inhibitor studies of MPO. A quinazoline compound, two phthalazine compounds and three benzodioxol compounds were identified as inhibitors of MPO [47,53], whereas two (phenylamino)acetic acid (F-PAAA and Cl-PAAA) compounds and two (phenylamino)acetic hydrazides (F-PAAH and Cl-PAAH) were previously recognized as potent EPO inhibitors [54]. Dopamine, which is a peroxidase substrate, was also examined in this study.

## 2. Material and Methods

### 2.1. Materials

Potassium bromide, phloroglucinol, dansylgycine and other chemicals were from Sigma unless stated otherwise and of the highest purity available. Hydrogen peroxide was from J.T. Baker™, Phillipsburg, New Jersey 8865. United States. Amplex™ UltraRed Reagent was from Thermo Fisher Scientific, New Zealand. 

### 2.2. Recombinant Human Peroxidasin 1 Variant (PXDN)

A previously characterized truncated peroxidasin variant, formerly annotated as hsPxd01-con4 [46], consisting of the four immunoglobulin domains and the peroxidase domain (amino acid residue 246-1314; numbering refers to full length PXDN including the signal peptide as shown in Uniprot Q92626), was used for all experiments using PXDN protein. Cloning, transient transfection, expression and the quality control of the purified recombinant PXDN was performed routinely, as described previously [39]. Molar concentrations are per heme and were determined using an extinction coefficient of 147,500 M^−1^ cm^−1^ for the heme Soret peak at 412 nm. For the conversion to mg, a molar mass of 140,000 g mol^−1^ was used and calculated from the molar concentration per heme. For simplicity we will refer to this variant as peroxidasin (PXDN) throughout the manuscript.

### 2.3. Inhibitors

The EPO inhibitors F-PAAA, Cl-PAAA, Cl-PAAH and F-PAAH were from Vitas-M Laboratory, Sales and Distribution Vitas M Chemical Limited 15F, Hong Kong and Enamine US Inc., NJ 08852, USA. Hydralazine-vanillin, quinazoline, lomefloxacin, thioridazine and metoclopramide were from Sigma. Benzodioxol 1, benzodioxol 2, benzodioxol 3, phthalazine 1 and phthalazine 2 were synthesized and the purity and quality were controlled by NMR spectroscopy, as described previously [47,54,55,56,57]. Structures of compounds are summarized in Table 1. Inhibitor stock solutions of 36 mM were made up in water and if insoluble, in 20–50% ethanol (*v*/*v*).

### 2.4. Dansylglycine Halogenation Activity Assay

Dansylglycine (DG) was made up fresh every day and kept in the dark at room temperature. A 15 mM stock solution was made up in ethanol and diluted 1:10 in phosphate buffer, before being further diluted to 50 μM in the halogenation activity assay (the residual ethanol concentration of 0.3% (*v*/*v*) did not affect the assay). We mixed 50 nM PXDN in 100 mM phosphate buffer pH 7.4 with 50 μM DG and 100 mM bromide, before the reaction was started by adding 200 μM of hydrogen peroxide to a final volume of 1.5 mL. Loss of fluorescence was monitored for 60 s using a F-7000 Hitachi (Methrom-Inula, Vienna) fluorometer (λ_ex_ 340 nm, λ_em_ 550 nm). To test the inhibition of halogenation activity, 24 μM of the respective inhibitor was added before the reaction was started with hydrogen peroxide. The loss of fluorescence was fitted linearly to determine the rate of reaction (min^−1^). The effect of the inhibitors was expressed as % inhibition, relative to the rate of the halogenation activity of PXDN without an inhibitor. IC_50_ values were determined by varying inhibitor concentrations (0–48 μM).

### 2.5. Amplex Oxidation Activity Assay in the Absence and Presence of Bromide

100 µL of 50 μM Amplex™ UltraRed and 20 μM hydrogen peroxide in 50 mM phosphate buffer pH 7.4 and the respective inhibitor at twice of its final concentration was added to a 96-well plate. The reaction was started by adding 100 µL of 2 µg mL^−1^ PXDN in 50 mM phosphate buffer pH 7.4, resulting in the final concentrations of 1 µg mL^−1^ of PXDN, 25 μM Amplex and 10 μM hydrogen peroxide. The plate was incubated in the dark for 15 min at 37 °C before the fluorescence of resorufin, the resulting Amplex oxidation product, was detected using a SpectraMax iD3 fluorescent plate reader from Molecular Devices LLC, San Jose, CA 95134 (λ_ex_ 544 nm, λ_em_ 590 nm, end point measurement). The same assay was also performed with 50 mM bromide present. To determine IC_50_ values, Amplex oxidation (measured as the end point as described above) was plotted versus the concentration of the inhibitor and expressed as % inhibition. For phloroglucinol, the inhibitor concentrations were lower than for the other inhibitors tested.

### 2.6. Stopped-Flow Spectroscopy

Pre-steady-state spectra were recorded with the stopped-flow apparatus SX.18MV (Applied Photophysics Ltd, Leatherhead, UK) connected to a diode array detector (DAD), with the first spectrum recorded 14 ms after the mixing of the reactants. Due to the inherent instability of Compound I of PXDN, the sequential mixing mode was used to generate Compound I. Equal volumes of 4 μM PXDN and 4 μM hydrogen peroxide were mixed in the aging loop [41,46]. After a delay time of 200 ms, an equal volume of the respective inhibitor was added, to give a final concentration of 1 μM Compound I and the indicated inhibitor concentrations. Interconversion of redox intermediates of PXDN was monitored for 60 s. The optical quartz cell had a volume of 20 μL and a path length of 10 mm. All reactions were performed in 100 mM phosphate buffer, pH 7.4, and at 25 °C. Time traces (410 nm to monitor Compound I and ferric PXDN, 432 nm for Compound II and 550 and 590 nm to monitor Compound III [46]) were extracted from the time-resolved spectra.

### 2.7. Reversibility of Inactivation of Human Peroxidasin

200 ng of PXDN in 50 μL PBS was added per well of a Corning high-binding ELISA microplate for 2 h at room temperature. After four PBS washes, the respective inhibitors were added at a final concentration of 20 μM in phosphate buffer pH 7.4, before 10 μM of hydrogen peroxide was added in a final volume of 50 μL. In a control experiment, no hydrogen peroxide was added. After 10 min of incubation, the plates were washed four times with PBS and activity was measured as previously described [29], by adding 50 μL/well of 50 μM Amplex™ UltraRed Reagent, 20 μM hydrogen peroxide and 50 mM bromide in 50 mM phosphate buffer pH 7.4. The plate was incubated for 30 min in the dark at 37 °C before fluorescence was measured (λ_ex_ 544 nm, λ_em_ 590 nm) and expressed as % activity of the untreated control.

### 2.8. Inhibition of Collagen IV Cross-Linking in Decellularized Extracellular Matrix and Cell Culture

PFHR9 cells (ATCC) were grown in Dulbecco’s Modified Eagle Medium (Gibco), supplemented with 10% heat-inactivated foetal bovine serum (Moregate) and 1% Penicillin Streptomycin (Gibco). As described previously [14], cells were seeded in 150 mm cell culture dishes and ECM was isolated. The concentration of the isolated ECM protein was measured (Direct Detect) and the yield was typically ~5 mg per dish. To test the inhibition of collagen IV cross-linking, the respective inhibitors (PHG, Cl-PAAA, Cl-PAAH, F-PAAH and benzodioxol 2) were added to 40 μL aliquots of ECM (containing ~300–350 μg protein). After 10 min, 100 μM bromide was added and the cross-link formation was started by adding 100 μM hydrogen peroxide. After 1 h at 37 °C, the reaction was stopped, by adding 1 mM sodium azide, and ECM was digested with 2 mg mL^−1^ of collagenase type I (Worthington) for 48 h at 37 °C. The digest was spun (20 kg, RT, 20 min) and the extent of cross-linking of the NCD of collagen IV was determined by the resolution of the supernatant protein on a 12% sodium dodecyl-sulfate polyacrylamide gel electrophoresis (SDS-PAGE). The inhibition of cross-link formation was visualized by Western blotting using standard procedures (primary antibody: Chondrex Rat alpha1 IV NCI mAb Clone H11 7070, 1:1000 dilution in 2% skim milk in TBST; Rabbit anti rat secondary Ab, 1:500 dilution). Densitometric analyses of both dimer bands (Alliance Uvitec Cambridge imager) were used to determine the extent of inhibition of the cross-link formation. For the inhibition of collagen IV cross-linking in cell culture, PFHR9 cells (ATCC) were seeded in 12-well plates at high density (~2 × 10^5^ cells per well). Once full confluence was reached, the medium was changed daily, and inhibitors were added at the indicated concentrations in 1 mL of medium for 7 days. The medium was removed, and cells were washed twice with PBS, before they were scraped in 200 µL of collagenase digestion buffer (50 mM HEPES pH 7.4 containing 10 mM calcium chloride, 0.1 mM benzamidine hydrochloride, 25 mM aminocaproic acid, 1 mM PMSF and 0.5 mg mL^−1^ of collagenase type I (Worthington). After an incubation of ~20 h at 37 °C, collagenase digest was centrifuged (20 kg, RT, 15 min) and an inhibition of cross-link formation was detected by Western blotting, as described above.

## 3. Results

### 3.1. Inhibition of Dansylglycine Halogenation Activity of Human Peroxidasin

Initially, we investigated the inhibition of halogenation activity of PXDN using the dansylglycine (DG) halogenation assay. DG is a fluorescent amino acid that reacts rapidly with HOBr (7.3 × 10^6^ M^−1^ s^−1^). It becomes brominated, which leads to loss of fluorescence in a concentration-dependent manner at an approximate stoichiometric ratio of 1:1 (Figure S1) [58].

**Figure 2:**
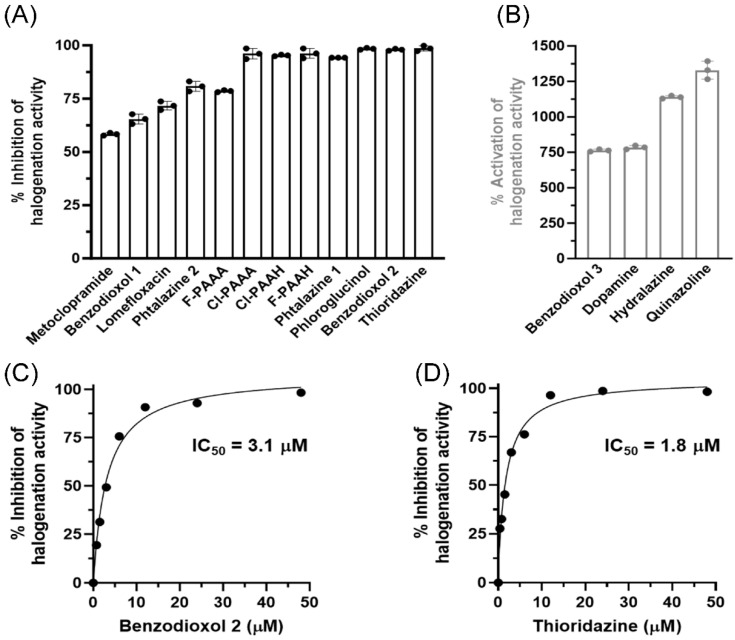
Effect of inhibitors on bromination activity of PXDN in dansylglycine assay. (**A**) Inhibition and (**B**) activation of bromination activity of PXDN. We mixed 50 nM PXDN, 50 μM DG, 24 μM inhibitor and 100 mM bromide in 100 mM phosphate buffer pH 7.4; the reaction was started by adding 200 μM hydrogen peroxide. Bars represent the mean of three individual measurements, depicted as single data points ±SD. (**C**,**D**) IC_50_ values for inhibition of PXDN bromination activity by benzodioxol 2 and thioridazine.

PXDN was mixed with DG in the presence of bromide before the reaction was started with hydrogen peroxide. A loss of fluorescence followed over time and the rate of reaction was determined in the absence and presence of the respective inhibitors. The reaction was dependent on both hydrogen peroxide and bromide, demonstrating that DG is not directly oxidized by PXDN with hydrogen peroxide alone. Twelve of the sixteen compounds tested inhibited hypobromous acid formation (Figure 2A). Phloroglucinol (PHG), benzodioxol 2 and thioridazine were identified as the most potent compounds, inhibiting 97–99% of PXDN bromination activity. The IC_50_ values for benzodioxol 2 and thioridazine were determined to be in the low micromolar range, as shown in Figure 2C,D. Interestingly, four of the compounds tested (hydralazine–vanillin, benzodioxol 3, quinazoline and the peroxidase substrate dopamine) strongly enhanced PXDN bromination activity (~7.5 to 13-fold) as shown in Figure 2B.

### 3.2. Inhibition of Amplex Oxidation Activity of Human Peroxidasin 

**Figure 3:**
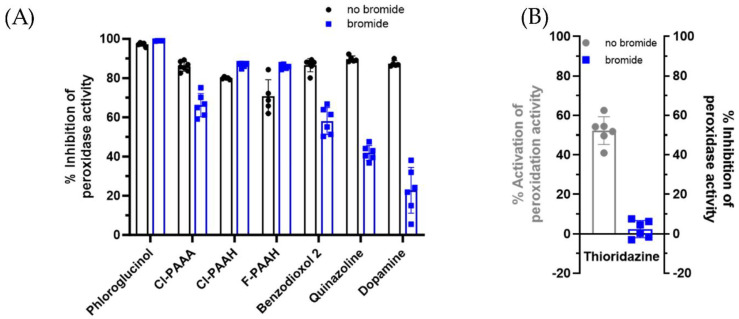
Effect of identified PXDN halogenation inhibitors and activators on Amplex oxidation. (**A**) Inhibition of Amplex oxidation by phloroglucinol, Cl-PAAA, Cl-PAAH, F-PAAH, benzodioxol 2, quinazoline and dopamine in the absence (black) and presence of bromide (blue). (**B**) Activation of Amplex oxidation by thioridazine in the absence (grey) and inhibition of Amplex oxidation in the presence of bromide (blue). 1 µg mL^−1^ PXDN, 20 μM inhibitor, 25 μM Amplex and 10 μM hydrogen peroxide without and with 50 mM bromide. Bars represent the mean of 4–6 independent experiments ±SD with each point depicting an individual data point.

To gain a better understanding of the mechanism of inhibition of PXDN, we investigated the inhibitory capacity of six of the most potent bromination inhibitors, plus the activators dopamine and quinazoline, in a peroxidase assay. Phthalazine 1 was excluded due to colorimetric interference with the assay. PXDN was added to a solution containing hydrogen peroxide and Amplex Ultrared, and the effect of each compound on the formation of the fluorescent product resorufin was measured. As depicted in Figure 3A, (black bars) phloroglucinol was the most potent inhibitor (>95% inhibition), with the others inhibiting by between 70 and 90%. Interestingly, thioridazine, which was a very potent bromination activity inhibitor, enhanced the oxidation of Amplex by about 50% (Figure 3B, grey bar).

### 3.3. Inhibition of Amplex Oxidation Activity of Human Peroxidasin in the Presence of Bromide

Amplex oxidation in the presence of bromide was also measured (Figure 3A,B, blue bars). In this assay, Amplex is oxidized both directly in the peroxidation cycle and by hypobromous acid, which is generated in the halogenation cycle. Bromide has been shown to increase Amplex oxidation in this assay, by approximately a factor of two [29]. The presence of bromide did not change the inhibition of Amplex oxidation by phloroglucinol (>95%). Inhibition by Cl-PAAH and F-PAAH was increased to 90%, whereas Cl-PAAA, benzodioxol 2, quinazoline and dopamine were less effective in the presence of bromide (between 70 and 25% inhibition). The activation of Amplex oxidation by thioridazine was mostly abolished when bromide was present, showing neither activation of Amplex oxidation, nor much inhibition (Figure 3B, blue bar). The oxidation of Amplex is reduced, due to the inhibitory effect of thioridazine on bromination.

**Figure 4:**
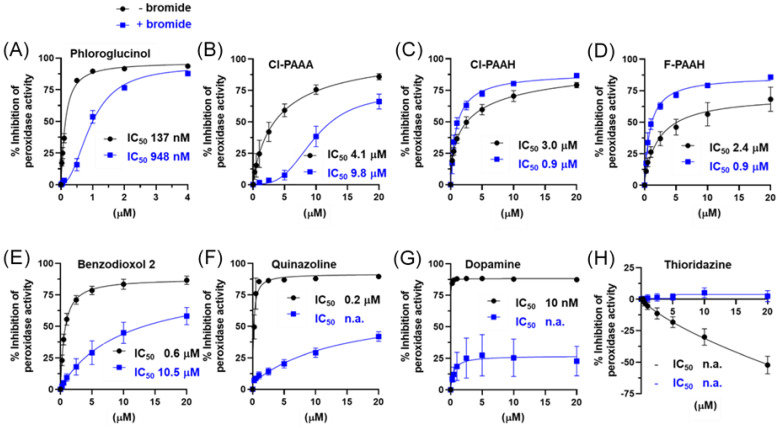
Concentration dependence of inhibition of Amplex oxidation activity in the absence and presence of bromide and respective IC_50_ values. Inhibition of Amplex oxidation activity of 1 µg mL^−1^ PXDN, 25 μM Amplex and 10 μM hydrogen peroxide either without (black) or with 50 mM bromide (blue) measured at various inhibitor concentrations. Points represent the mean of four to six individual measurements ±SD. Curves were fitted and IC_50_ values were determined in Graphpad prism using inhibitor vs. response, variable slope (four parameters) best-fit values. n.a. indicates where inhibition did not reach 50% at 20 μM inhibitor.

The IC_50_ values for the Amplex assay in the absence and presence of bromide were determined as depicted in Figure 4A–H (black without and blue with bromide) and are summarized in Table 2. In the absence of bromide, the IC_50_ values were lowest for phloroglucinol, dopamine and quinazoline. However, in the presence of bromide, inhibition was reduced to some degree for phloroglucinol and much more for dopamine and quinazoline. Cl-PAAA, Cl-PAAH, F-PAAH and benzodioxol 2 were inhibited at low micromolar concentrations in the absence and presence of bromide.

### 3.4. Reversibility of Inhibition of Human Peroxidasin

**Figure 5:**
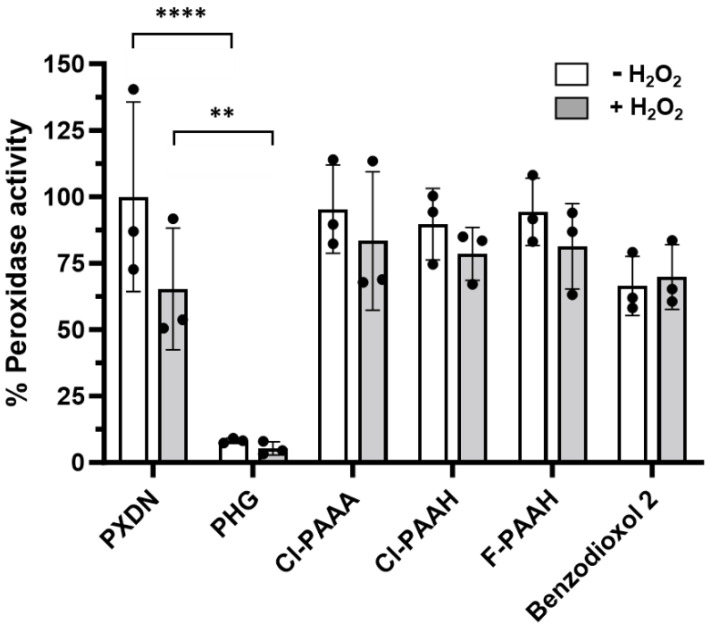
Reversibility of inhibition of PXDN by phloroglucinol (PHG), Cl-PAAA, Cl-PAAH, F-PAAH and benzodioxol 2. 200 ng of immobilized PXDN was treated with 20 μM of the respective inhibitor in the absence (white bars) or presence (grey bars) of 10 μM hydrogen peroxide for 10 min, before plate was washed four times with PBS and activity of PXDN was measured with Amplex. Bars represent the mean of three independent experiments ± SD. **** *p* < 0.0001, ** *p* < 0.0038 using one-way ANOVA analysis followed by Sidak’s multiple comparisons.

To test for the reversibility of inhibition, PXDN was immobilized on a high binding plate, incubated with inhibitor without and with hydrogen peroxide, then washed before analysing in the Amplex assay. Only phloroglucinol still resulted in more than 95% inhibition (*p* < 0.0001) whereas Cl-PAAA, Cl-PAAH, F-PAAH and benzodioxol 2 showed no statistically significant difference between untreated PXDN or PXDN that was only preincubated with hydrogen peroxide (Figure 5). This confirms that the latter compounds are reversible inhibitors. In contrast, phloroglucinol inhibited PXDN irreversibly, either by tightly binding to the active site or by covalent adduct formation; further studies are required to elucidate the nature of this inhibition. Interestingly, pre-incubation of PXDN with hydrogen peroxide and phloroglucinol was not required for the irreversible inhibition of PXDN by phloroglucinol.

### 3.5. Stopped-Flow Spectrophotometry of Reaction of Human Peroxidasin Compound I with Inhibitors Radical Product

**Figure 6:**
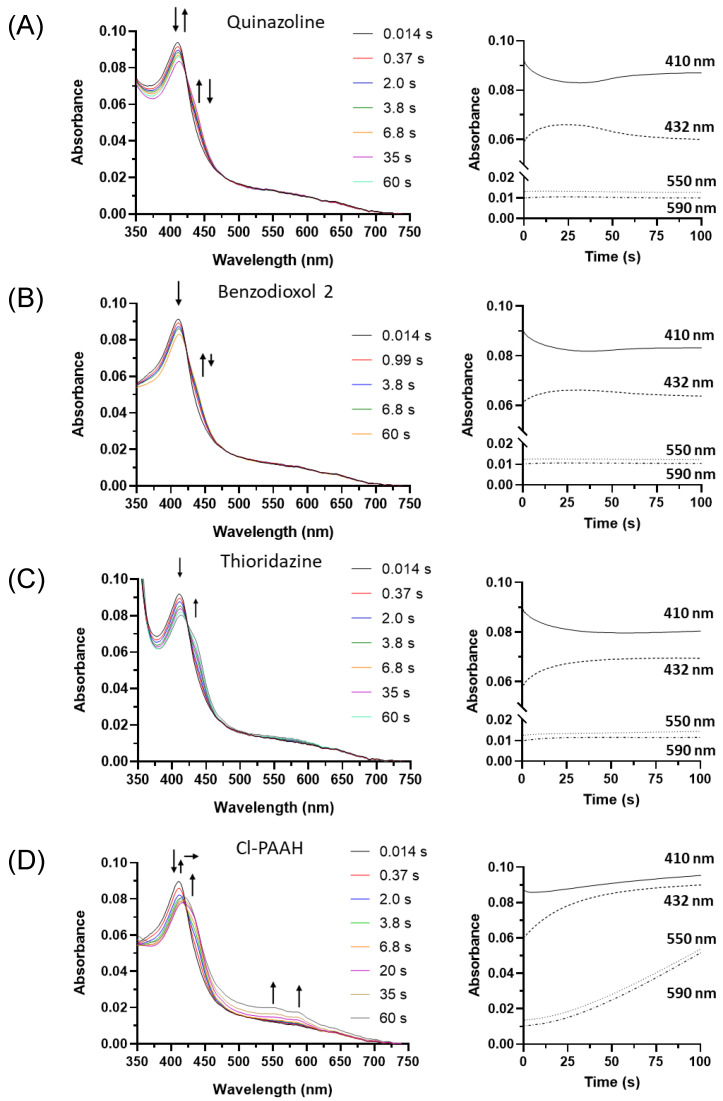
Stopped-flow spectra (left panels) and time traces (right panels) of PXDN Compound I reacted with quinazoline, benzodioxol 2, thioridazine and Cl-PAAH. Spectra of 1 μM of PXDN Compound I reacted with (**A**) 50 μM of quinazoline, (**B**) 100 μM benzodioxol 2, (**C**) 100 μM thioridazine and (**D**) 100 μM Cl-PAAH. Colour-coded time resolved spectra were recorded at indicated times (left panels) and spectral changes over time at 410, 432, 550 and 590 nm are depicted in 6A–D (right panels). Arrows indicate direction of changes.

Sequential stopped-flow spectrophotometry was used to study the redox intermediates involved when PXDN Compound I was reacted with a selection of inhibitors and dopamine. Spectral changes were recorded (left panels, Figure 6A–D and Figure S2A–D) and time traces of redox intermediates were extracted from the spectra (right panels, Figure 6A–D and Figure S2A–D). The conversion of redox intermediates followed, at 410 nm for Compound I and ferric enzyme, 432 nm for Compound II and 550 nm and 590 nm for Compound III. Four types of mechanisms of inhibition were determined from the redox intermediates observed. Group one comprised good peroxidation substrates. These show a transient rapid increase in Compound II, before reversion to the native enzyme, and include quinazoline (Figure 6A), hydralazine and dopamine (Figure S2A,B). Their activation of PXDN bromination activity can be explained by resolving Compound II back to the ferric enzyme. Group two comprised poor peroxidation substrates. These show the rapid formation of Compound II and slow (benzodioxol 2; Figure 6B) or negligible (thioridazine; Figure 6C) conversion back to ferric enzyme. These are good substrates for Compound I and poor substrates for Compound II. They are effective at blocking bromination activity by trapping the enzyme at Compound II. Group three were substrates that formed Compound III, as characterised by the increase in absorbance at 550 and 590 nm and a shift of the heme Soret peak from 412 nm to 425 nm. This group comprises Cl-PAAH as depicted in Figure 6D, and Cl-PAAA and F-PAAH as shown in Figure S2C,D.

**Figure 7:**
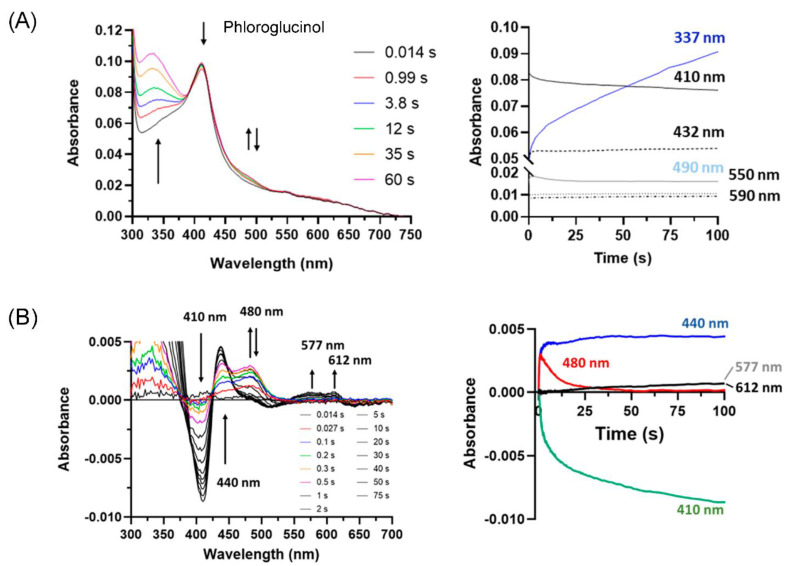
Stopped-flow spectra (left panels) and time traces (right panels) of PXDN Compound I reacted with phloroglucinol. (**A**) Spectra of 1 μM of PXDN Compound I reacted with 100 μM phloroglucinol. Colour-coded time resolved spectra were recorded at indicated times (left panel) and spectral changes over time at 410, 432, 550, 590 nm and additionally 337 nm (blue) and 490 nm (grey) are depicted in (**A**) (right panel). Arrows indicate direction of changes. (**B**) Difference spectra of PXDN Compound I reacted with phloroglucinol. Same spectra as in (**A**) depicted as difference spectra (first recorded spectrum deducted from all spectra). Colour-coded time resolved spectra were recorded at indicated times (s) (left panel) and spectral changes over time at 410, 440, 480, 577 and 612 nm are shown in the right panel. Arrows indicate direction of changes.

Spectral changes with phloroglucinol do not fit into any of these three groups, implying a different mechanism of inhibition. As depicted in Figure 7A, there were only minimal spectral changes, with the most pronounced being an increase at 337 nm. This represents the formation of an oxidation product of phloroglucinol that was rapid for ~1 s of reaction time, then slowed to become enzyme-independent and light-induced. The slower increase was not observed if the reaction was not exposed to the diode array detector (as investigated under steady-state conditions). Better visualisation of the spectral changes with phloroglucinol was obtained from difference spectra depicted in the left panel of Figure 7B, and the extracted time traces of minima and maxima (right panel). PXDN showed a loss of absorbance at 410 nm, concomitant with an increase at 440 nm. Both of these spectral changes did not revert back. Additionally, there is a fast increase at 480 nm over 1 s, followed by a slower decay and small increases at 577 and 612 nm. These spectral changes are not due to the formation of Compound II (maximum 432 nm) or Compound III (maxima at 550 nm and 590 nm, as in Figure 6D). The distinct spectral change at 440 and 480 nm indicated the formation of an adduct to either the heme prosthetic group or an amino acid residue of the active site. The increase in absorbance at 577 and 612 nm further confirmed changes in the active site. Phloroglucinol could either act as a tight binding ligand or a suicide substrate with the radical product (formed when reacting with Compound I) covalently attached to the heme group or an active site amino acid residue.

### 3.6. Inhibition of Human Peroxidasin Activity in Decellularized Extracellular Matrix

**Figure 8:**
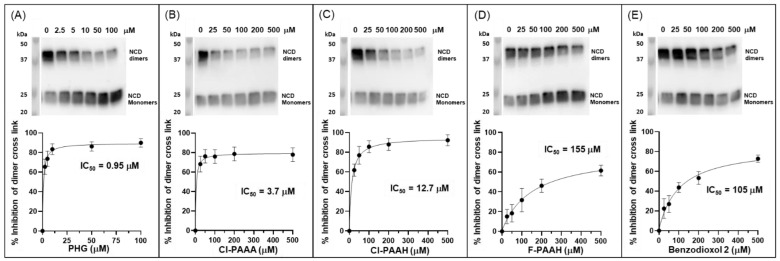
Inhibition of collagen IV NCD sulfilimine cross-link formation in isolated ECM by (**A**) phloroglucinol, (**B**) Cl-PAAA, (**C**) Cl-PAAH, (**D**) F-PAAH and (**E**) benzodioxol 2. Isolated ECM from PFHR9 cells was incubated with the respective inhibitor at increasing concentrations, and collagen IV NCD dimer formation was determined by Western blotting after collagenase digestion (top panels, representative blot of three independent experiments). Densitometry was used to quantify the inhibition of collagen IV NCD dimer formation (quantification of dimer bands relative to untreated control in lane 1, A–E), which was plotted against inhibitor concentrations (n = 3). The curve was fitted and IC_50_ values were determined in Graphpad prism using inhibitor vs. response, variable slope (four parameters) best-fit values.

The most promising inhibitors were tested for their ability to inhibit PXDN activity in decellularized ECM by measuring the inhibition of collagen IV NCD sulfilimine cross-link formation. The ECM was isolated from PFHR9 cells grown in the presence of phloroglucinol to inhibit cross-link formation during cell growth. Phloroglucinol was previously presumed to act as a reversible inhibitor and to wash away fully during ECM isolation and washing steps [4,12,14,59], restoring PXDN activity. However, if phloroglucinol acts as an irreversible inhibitor, it may only be a residual activity that is detected in the ECM preparation. Inhibitors were added to aliquots of isolated ECM, before bromide and hydrogen peroxide were added to start cross-link formation. After the digestion of ECM with collagenase, collagen IV cross-linking was visualized by Western blotting, and densitometry was used to quantify the extent of the inhibition of cross-link formation, to determine IC_50_ values for this ECM model. Phloroglucinol was the most potent inhibitor, with an IC_50_ of about 950 nM (Figure 8A). Cl-PAAA and Cl-PAAH also inhibited, with IC_50_ values of 3.7 μM and 12.7 μM, respectively (Figure 8B,C). F-PAAH and benzodioxol 2 were much less potent, with IC_50_ values of 155 μM and 105 μM, respectively (Figure 8D,E).

### 3.7. Inhibition of Human Peroxidasin Activity in Cell Culture

**Figure 9:**
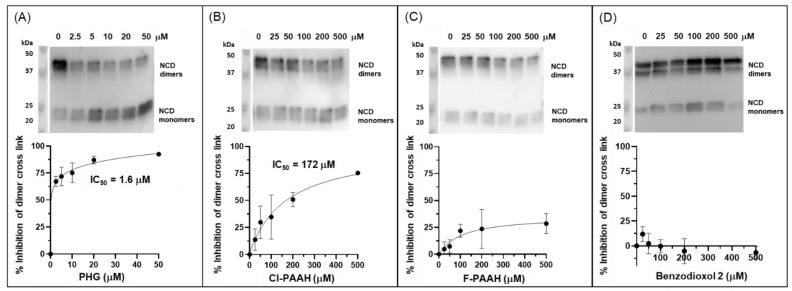
Inhibition of collagen IV NCD sulfilimine cross-link formation of PFHR9 cells by (**A**) phloroglucinol, (**B**) Cl-PAAH, (**C**) F-PAAH and (**D**) benzodioxol 2. PFHR9 cells were grown in the presence of the respective inhibitor at increasing concentrations and collagen IV NCD dimer formation was determined by Western blotting after collagenase digestion (top panels, representative blot of three independent experiments). Densitometry was used to quantify inhibition of collagen IV dimer formation (quantification of dimer bands relative to untreated control in lane 1, **A**–**D**), which was plotted against inhibitor concentrations (n = 3). A curve was fitted and IC_50_ values were determined in Graphpad prism using inhibitor vs. response, variable slope (four parameters) best-fit values.

To test the inhibition of PXDN in a more physiological setting, we included the inhibitors in the medium during the growth of PFHR9 cells and tested their ability to inhibit collagen IV cross-linking. Phloroglucinol performed best, with an IC_50_ value of 1.6 μM (Figure 9A). Cl-PAAA, which was inhibiting collagen IV cross-linking in decellularized ECM quite potently, was cytostatic or cytotoxic at concentrations >100 μM and, therefore, excluded from this experiment. Cl-PAAH (Figure 9B) displayed an IC_50_ value of about 172 μM, and F-PAAH and benzodioxol 2 were ineffective (Figure 9C,D). Again, these experiments highlight the effectiveness of phloroglucinol in inhibiting PXDN activity in cell culture at low micromolar levels.

## 4. Discussion

Human heme peroxidases play fundamental roles in host defence, thyroid hormone synthesis and basement membrane stabilization. Their ability to generate highly reactive oxidants [11] underlie many pathologies and has driven the search for peroxidase inhibitors. Potent inhibitors have been identified; however, due to high amino acid sequence homology and similar active site architecture, less specific inhibitors may not just inhibit one peroxidase. In the present study, we tested the inhibition of PXDN activity by potent MPO and EPO inhibitors, with the aim to identify lead compounds for specific PXDN inhibitor design and to elucidate their mechanism and efficacy of inhibition.

Comparative sequence analysis confirms that all active site amino acid residues critical for catalytic activity and structural integrity are highly conserved in PXDN, including the His, Arg and Gln triad and the calcium-binding residues on the distal site and the His and Gln on the proximal site [38]. Like in EPO and LPO, the heme is covalently attached to the protein via two ester bonds between the aspartate (D826) and glutamate (E980) residues and the 5- and 1-methyl groups of the heme. This results in a similar redox potential of the Fe(III)/Fe(II) couple (LPO −176 mV, PXDN −128 mV and EPO −126 mV) [46,60,61], inferring comparable reactivity with inhibitors.

Firstly, we found that 12 of the 15 inhibitors were medium to highly effective at inhibiting the bromination activity of PXDN in the absence of a peroxidase substrate (Figure 2A). The IC_50_ values of the two most potent inhibitors of bromination were in the low micromolar range (Figure 2C,D), which is sufficiently low for pharmacological inhibition. The low IC_50_ values also support the concept that the loss of signal was due to the inhibition of PXDN and not due to the inhibitorscavenging HOBr. Apart from phloroglucinol, these compounds acted by reacting with Compound I to divert PXDN from the halogenation to the peroxidation cycle by forming Compound II (Figure 1, reaction 3), or by forming Compound III and trapping PXDN outside the halogenation cycle. If Compound II turnover was slow (reaction 4), bromination was inhibited. Interestingly, four compounds, including the peroxidase substrate dopamine, potently enhanced bromination (Figure 2B) by facilitating the rapid turnover of Compound II and allowing PXDN to re-enter the halogenation cycle via Compound I. The six most potent inhibitors and two bromination activators were tested for their ability to inhibit peroxidation activity (Figure 3 and Figure 4) in an assay, with Amplex as the peroxidase substrate. The two bromination activators, quinazoline and dopamine, were highly effective inhibitors in this assay, by outcompeting the Amplex for both Compound I and II. Thioridazine, one of the most effective bromination inhibitors, activated peroxidase activity, indicating that it may act as a better substrate for Compound I than Amplex and therefore speed up the peroxidation cycle. All other tested compounds had IC_50_ values in the low micromolar range. The addition of bromide to the Amplex assay gives a system that measures a combination of bromination and peroxidase activity. Five compounds were potent inhibitors in this assay, namely phloroglucinol, Cl-PAAA, Cl-PAAH, F-PAAH and benzodioxol 2. Thioridazine, dopamine and quinazoline became ineffective, as expected of bromination activators.

Multi-mixing stopped-flow spectrophotometry was used to investigate the redox intermediates involved in inhibition (Figure 6 and Figure S2). We identified four different categories, in line with the above observations of the inhibition of bromination and Amplex oxidation. Firstly, compounds that are very good substrates for Compound I and II (i.e., quinazoline and dopamine, Figure 6A and Figure S2A) enhanced bromination, due to reverting any Compound II back to the ferric form and strongly inhibiting Amplex oxidation in the absence of bromide; however, they became ineffective in the presence of bromide. Secondly, poor Compound II substrates (e.g., benzodioxol 2, Figure 6B) delayed the reduction of Compound II to a ferric enzyme, and thereby inhibited both the halogenation and peroxidation cycles reversibly. Thioridazine did not react with Compound II at all, trapping PXDN highly effectively in bromination-inactive Compound II (Figure 6C), which was lost with Amplex, making this Compound II trapper ineffective for PXDN inhibition. Thirdly, the third category of inhibitors converted PXDN to the bromination- and peroxidation-inactive Compound III (Cl-PAAA, Cl-PAAH and F-PAAH, Figure 6D and Figure S2C,D). This is in line with these compounds potently inhibiting EPO by converting it to Compound III, highlighting the high active site homology and similar reactivity of PXDN and EPO [54]. (iv) Phloroglucinol stood out as the most potent inhibitor in all the assays, with IC_50_ values in the submicromolar range. It was also the only one that inhibited irreversibly. Spectral measurements showed that it inhibited without forming Compound II or III. This could either be due to mechanism-based inhibition or tight binding to the active site that did not dissociate within the timeframe of the experiment. However, the spectral changes are more supportive of adduct formation. (Figure 7). Although irreversible inhibition was not dependent on preincubation with hydrogen peroxide and phloroglucinol, it is possible that sufficient peroxide was generated through autoxidation [42], or that inactivation occurred when it was added to start the activity assay. A mechanism-based inhibition was described for LPO and TPO for resorcinol derivatives including phloroglucinol, although hydrogen peroxide was strictly required for inhibition. The spectral changes described for LPO are similar to what we observed using stopped-flow spectroscopy [52]. Detailed understanding of the molecular mechanism of inhibition of PXDN by phloroglucinol still needs further investigation. MPO was shown not to be inhibited by phloroglucinol and resorcinol derivatives. Similarly, MPO inhibitors tested in this study were less effective than EPO inhibitors, most likely due to differences in structure, redox potential and reactivity [61].

When testing the efficacy of collagen IV NCD sulfilimine link formation in an ECM model, we observed potent inhibition by phloroglucinol (IC_50_ value of 0.95 μM) and Cl-PAAA and Cl-PAAH showing promise, with IC_50_ values of 3.7 and 12.7 μM, respectively (Figure 8). When this selection of inhibitors was tested under cell culture conditions, only phloroglucinol showed high efficacy, with an IC_50_ value of 1.6 μM, while the other inhibitors became ineffective (Figure 9). The phloroglucinol value is similar to the previously published value of 0.5 μM, for the inhibition of collagen IV cross-linking [3]. Phloroglucinol is well tolerated in cell culture and does not impact cell proliferation. It is a small and highly hydrophilic compound, whereas Cl-PAAA, Cl-PAAH, F-PAAH and benzodioxol 2 are more hydrophobic, and may be ineffective in cell culture due to off-target binding.

## 5. Conclusions

Using a semi-targeted approach of testing EPO and MPO inhibitors, we have identified potent inhibitors of PXDN that may be useful leads for the design of compounds with greater specificity. Our work has highlighted that most inhibitors are not specific to just one peroxidase, which has important clinical implications. According to the context, it may be beneficial to inhibit more than one peroxidase or only one, but highly specifically. Although phloroglucinol was the most effective inhibitor, it also potently inhibits LPO and TPO [52]. It would be advantageous, therefore, if compounds based on phloroglucinol could be developed with greater specificity. Vice versa, methimazole, a TPO inhibitor used in the clinic to treat hyperthyroidism, also potently inhibits collagen IV cross-link formation by PXDN [3].

We have established a simple Amplex–bromide combination assay to test the inhibition of PXDN peroxidase and halogenation activity, which is fit for high-throughput screening of compounds of interest or drug library testing. We have also shown that ECM and cell culture models can be used to test efficacy under more physiological conditions. We further revealed that phloroglucinol inhibits PXDN irreversibly and is currently the best inhibitor to use in pathological models. Although other inhibitors are reversible, this is not necessarily a disadvantage physiologically, and they have potential for further development.

## Figures and Tables

**Table 1:** Abbreviation, name, peroxidase-inhibited (blue), structure and molecular weight (MW) of tested compounds.

Compounds	Structure	MW
**PHG** Phloroglucinol 1,3,5-Trihydroxybenzene PXDN, LPO, TPO	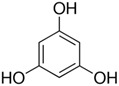	126.11
**Lomefloxacin** MPO	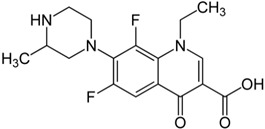	351.35
**Thioridazine** MPO	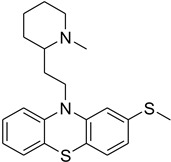	370
**Metoclopramide** MPO	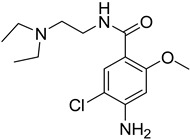	299
**Hydralazine-vanillin** (E)-2-methoxy-4-((2-phtalazine-1yl)hydrazono)methyl)phenol MPO	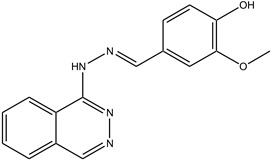	294
**Quinazoline** 2-(7-methoxy-4-methylquinazoline-2-yl)guanidine MPO	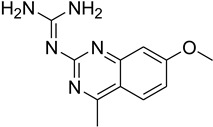	231
**Phthalazine 1** (E)-N,N-dimethyl-4-((2-(phthalazine-1-yl)hydrazono)methyl)aniline MPO	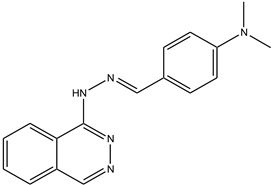	291.35
**Phthalazine 2** (E)-2-(2-(phtalazine-1-ly)hydrazono)ethanol MPO	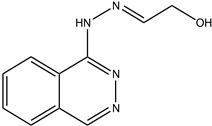	202
**Benzodioxol 1** 4-(1,3-benzodioxol-5-yloxy)butan-1-amine MPO	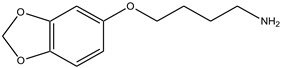	209.2
**Benzodioxol 2** 4-(1,3-benzodioxol-5-yloxy)batan-1,4-dimethylpiperazine MPO	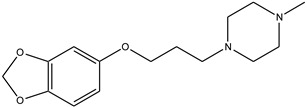	278.3
**Benzodioxol 3** 4-(4-Amino-butoxy)-benzene-1,2-diol MPO	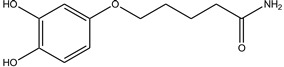	225.2
**F-PAAA** N-[(2-fluorophenyl)amino] acetic acid EPO	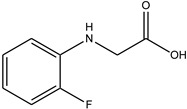	169.1
**Cl-PAAA** (2-[(4-chlorophenyl) amino]- acetic acid) EPO	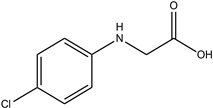	185.02
**Cl-PAAH** (2-[(4-chlorophenyl) amino]- acetohydrazide) EPO	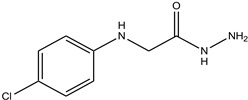	199.05
**F-PAAH** 2-[(2-fluorophenyl)amino]- acetohydrazide EPO	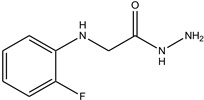	183.08
**Dopamine** Peroxidase substrate	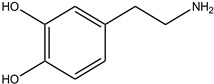	153.18

**Table 2:** Summary of compounds, IC_50_ values and mechanism of inhibition. n.d.—not determined and n.a.—not applicable, as 50% of inhibition was not reached.

Compound	IC_50_ Amplex Oxidation (μM)	IC_50_ Amplex Oxidation + Bromide (μM)	IC_50_ Halogenation Dansylglycin (μM)	IC_50_ DECM (μM)	IC_50_ Cells (μM)	Mechanism or Reaction Steady and Pre-Steady State Spectrophotometry	Irreversible Inhibition
Phloroglucinol	0.137	0.948	n.d.	0.95	1.6	Adduct formation or tight ligand binding	yes
Cl-PAAA	4.1	9.8	n.d.	3.7	toxic	forms Compound III	no
Cl-PAAH	3.0	0.9	n.d.	12.7	172	forms Compound III	no
F-PAAH	2.4	0.9	n.d.	155.2	n.a.	forms Compound III	no
Benzodioxol 2	0.6	10.5	2.4	104.6	n.a.	Substrate for CI, poor substrate for CII	no
Dopamine	0.010	n.a.	n.d.	n.a.	n.a.	Good substrate for CI and CII	n.a.
Quinazoline	0.2	n.a.	n.d.	n.a.	n.a.	Good substrate for CI and CII	n.a.
Thioridazine	n.a.	n.a.	1.5	n.a.	n.a.	Good substrate for CI, not a substrate for CII	n.a.

## Data Availability

Data is contained within the article or supplementary material.

## Supplementary Materials

The following supporting information can be downloaded at: https://www.mdpi.com/article/10.3390/antiox13010023/s1. Figure S1: (A) Dansylglycine bromination activity of PXDN in the presence of increasing concentrations of thioridazine. (B) Determination of stoichiometric ratio of hypobromous acid reacting with dansylglycine. Figure S2: Stopped-flow spectra (left panels) and time traces (right panels) of PXDN Compound I reacting with dopamine, hydralazine, Cl-PAAA and F-PAAH.

## Abbreviations

PXDN, human peroxidasin; EPO, eosinophil peroxidase; MPO, myeloperoxidase; LPO, lactoperoxidase; DG, dansylglycine; PHG, phloroglucinol; ECM, extracellular matrix; BM, basement membrane; NCD, non-collagenous domain of collagen IV; HOBr, hypobromous acid.
