# Supplementary material for: Peroxidasin Inhibition by Phloroglucinol and Other Peroxidase Inhibitors
Source: Antioxidants (Basel). 2023 Dec 21;13(1):23. doi: 10.3390/antiox13010023 (PMC10812467; doi:10.3390/antiox13010023)
Supplement: Supplementary file 1 [file antioxidants-13-00023-s001.zip › antioxidants-2745707-supplementary.pdf]

Supplementary data

Peroxidasin Inhibition by Phloroglucinol and Other Peroxidase Inhibitors

Martina Paumann-Page [1,2,*], Christian Obinger [2], Christine C. Winterbourn [1] and Paul G. Furtmüller [2,*]

[1] Mātai Hāora Centre for Redox Biology and Medicine, University of Otago Christchurch, Ōtautahi Christchurch 8011, New Zealand; christine.winterbourn@otago.ac.nz
[2] Institute of Biochemistry, Department of Chemistry, University of Natural Resources and Life Sciences, Vienna, Muthgasse 18, 1190 Vienna, Austria; christian.obinger@boku.ac.at
* Correspondence: martina.paumann-page@otago.ac.nz (M.P.-P.); paul.furtmueller@boku.ac.at (P.G.F.);
Tel.: +64-3-364-1559 (M.P.-P.); +43-1-47654-77277 (P.G.F.)

Keywords: peroxidasin; inhibition of catalytic activity; phloroglucinol; peroxidase inhibitors; hypobromous acid; oxidative stress; myeloperoxidase; eosinophil peroxidase; lactoperoxidase;
thyroid peroxidase

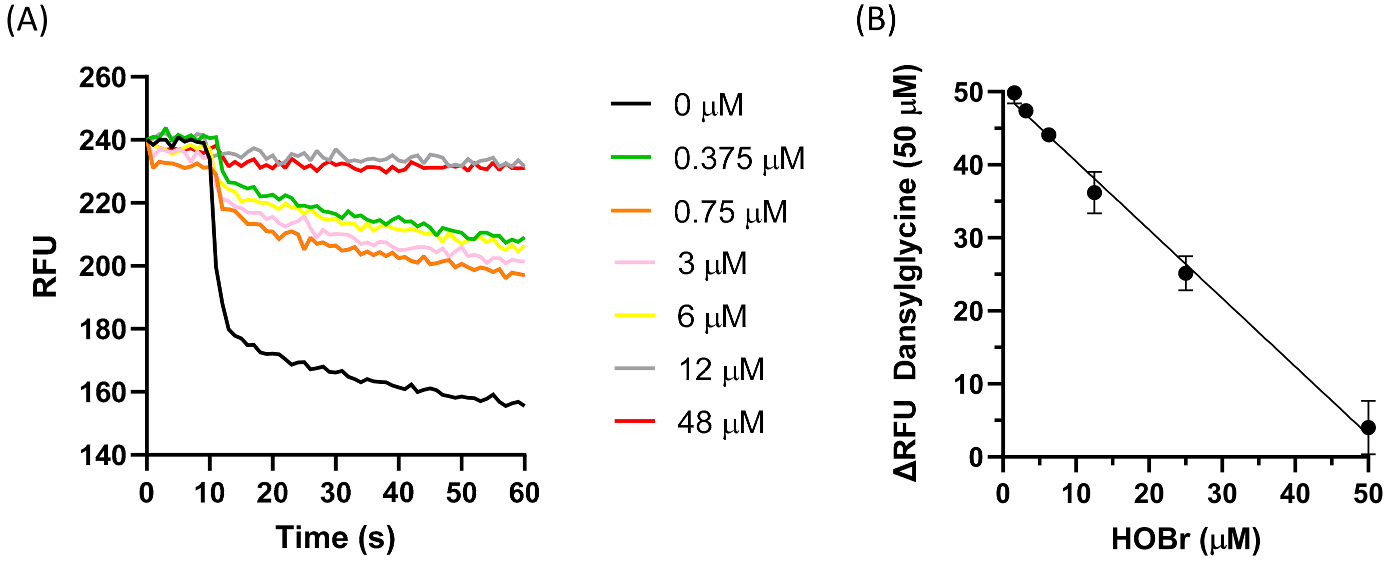

Figure S1: (A) Dansylglycine bromination activity of PXDN in the presence of increasing concentrations of thioridazine. 50 nM PXDN in 100 mM phosphate buffer pH 7.4, 50 μM DG, 100 mM bromide with 0, 0.375, 0.75, 3, 6, 12, 48 μM thioridazine present, before the reaction was started addition of 200 μM hydrogen peroxide. Loss of fluorescence due to HOBr generation was monitored over time (λ_{ex} 340 nm, λ_{em} 550 nm) for 60 s using a Hitachi (Inula, Vienna) fluorometer. Loss of fluorescence was fitted linearly to determine the rate of reaction (min^{-1}). **(B) Determination of stoichiometric ratio of hypobromous acid reacting with dansylglycine.** Loss of fluorescence was measured as endpoint at 10 min after 50 μM dansylglycine reacted with increasing amounts of hypobromous acid in 100 mM phosphate buffer pH 7.4. A ~1:1 ratio was determined.

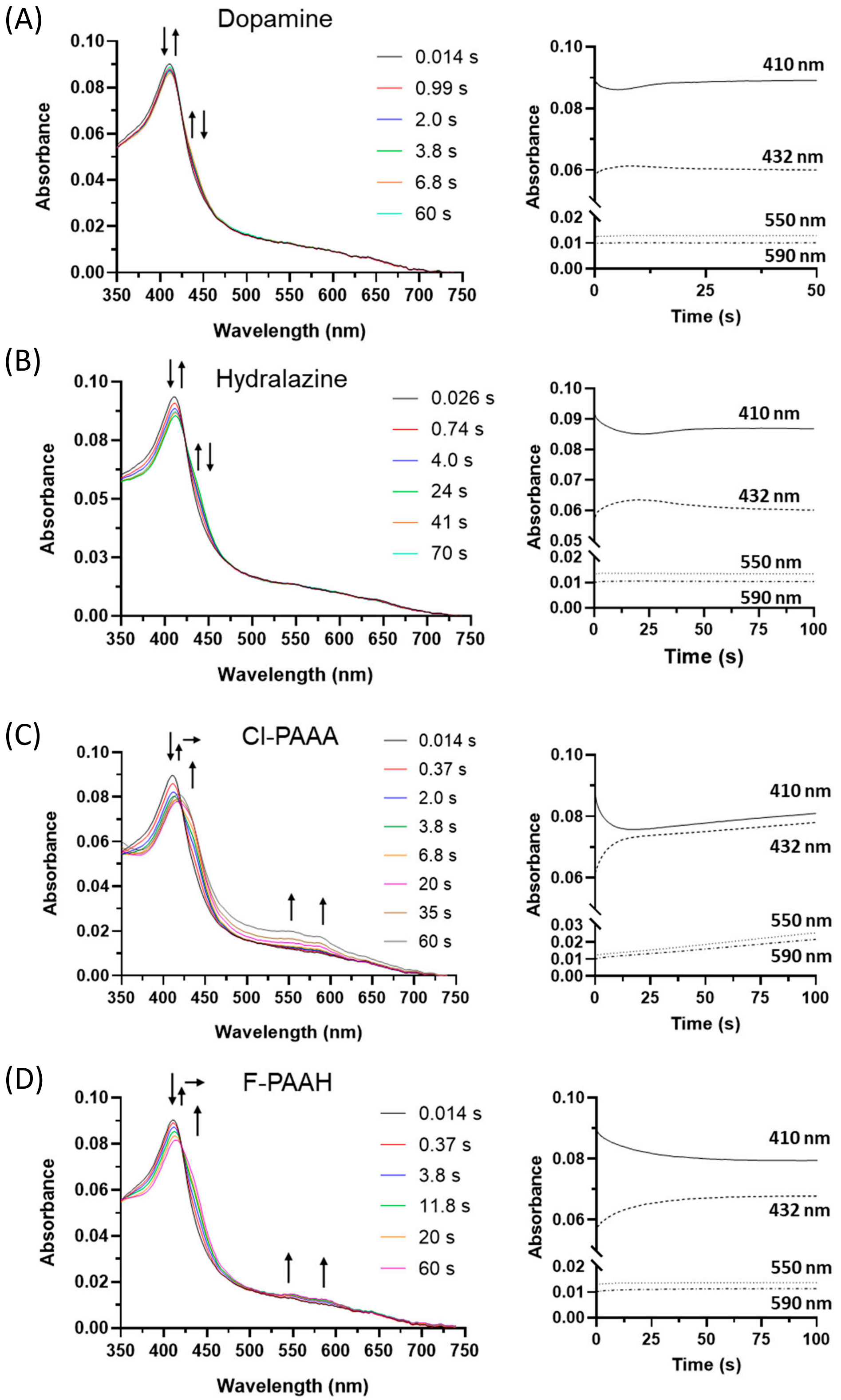

Figure S2: Stopped-flow spectra (left panels) and time traces (right panels) of PXDN Compound I reacting with dopamine, hydralazine, Cl-PAAA and F-PAAH. Spectra of 1 μM of PXDN Compound I reacting with (A) 10 μM of dopamine, (B) 10 μM hydralazine, (C) 100 μM Cl-PAAA and (D) 100 μM F-PAAH. Colour-coded time resolved spectra were recorded at indicated times (s) (left panels) and spectral changes over time at 410, 432, 550, 590 nm are depicted in the right panels. Arrows indicate direction of changes.
